# Enhancing translational researchers’ ability to collaborate with community stakeholders: Lessons from the Community Engagement Studio

**DOI:** 10.1017/cts.2018.323

**Published:** 2018-10-31

**Authors:** Yvonne A. Joosten, Tiffany L. Israel, Amy Head, Yolanda Vaughn, Victoria Villalta Gil, Charles Mouton, Consuelo H. Wilkins

**Affiliations:** 1 Department of Medical Education and Administration, Vanderbilt University School of Medicine, Nashville, TN, USA; 2 Vanderbilt Institute for Medicine and Public Health, Nashville, TN, USA; 3 University of Tennessee School of Social Work, Nashville, TN, USA; 4 Neighborhoods Resource Center, Nashville, TN, USA; 5 Meharry Vanderbilt Alliance, Nashville, TN, USA; 6 Department of Family Medicine, University of Texas Medical Branch, Galveston, TX, USA; 7 Department of Internal Medicine, Meharry Medical College, Nashville, TN, USA

**Keywords:** Community stakeholders, community engagement, patient engagement, community engagement studio, translational researchers

## Abstract

Community engagement is considered essential to effectively translate research into practice and is increasingly recognized as a key to successful clinical trial recruitment. Challenges to engaging community stakeholders in research persist and new methods are needed to facilitate meaningful stakeholder involvement. The Community Engagement Studio (CE Studio), a consultative model, has been used at every stage of the research process. Best practices drawn from the model could inform other methods of engagement. Using a mixed-methods approach that included evaluation surveys, impact surveys and interviews, we assessed the CE Studio program. We analyzed data from 75 CE Studios; 65 researchers and 591 community members completed surveys and 10 researchers completed interviews. Surveys indicate that 100% of researchers would request a CE Studio in the future, and 99.3% of community members would participate in a CE Studio again. We identified 6 practices to enhance community engagement in clinical and translational research: early input, researcher coaching, researcher humility, balancing power, neutral facilitator, and preparation of community stakeholders. These best practices may enhance the quality of existing community engagement approaches and improve the effectiveness of translational researchers’ efforts to engage community stakeholders in their work.

## Introduction

The recognition of patient and community engagement as vital to successful clinical trials recruitment [1] and translation of research into practice has heightened the need for effective mechanisms to involve patients, consumers, and other stakeholders in research [2–5]. Approaches to engage community stakeholders include interviews, focus groups, working groups, surveys, town hall meetings, nominal group techniques, and community listening sessions. However, there are many barriers to implementing these approaches including researchers’ lack of understanding and experience with engaging community stakeholders [6–11].

Programs intended to increase community engagement in research must accommodate researchers with varying levels of skill and experience engaging stakeholders, demonstrate the value of community input and address the barriers that prevent or derail meaningful community engagement (e.g., trust, bi-directional communication, power differences, scheduling conflicts, and compensation).

The Community Engagement Studio (CE Studio) [12] is a method that helps to overcome many of the common barriers to engagement and can be used by researchers regardless of prior experience or training in community engagement. The CE Studio incorporates key principles of community engagement including bi-directional communication, co-learning, a focus on community assets rather than deficits, and mutual benefit [9, 10, 13, 14]. Previous publications have described the CE Studio model process and use for specific projects [15, 16]. In this article, we describe best practices drawn from the development, refinement, and evaluation of the CE Studio model and implications for preparing clinical and translational researchers to effectively engage patients and other community stakeholders in their work.

### The CE Studio Process

The CE Studio is a consultative model that allows researchers to obtain project-specific input from patients and other community stakeholders. Developed by the Meharry-Vanderbilt Community Engaged Research Core (CERC), the CE Studio can be used in all phases of the research process to increase the relevance or patient-centeredness of the work, to respond to funder requirements for community input, or to address challenges in an active project such as participant recruitment or implementation. The CE Studio includes: (1) consultation with the researcher by the CE Studio team; (2) creation of a unique panel of community stakeholders or experts who represent the researcher’s population of interest; (3) pre-meeting coaching for the researcher by the CE Studio team; (4) orientation and preparation of the expert panel; (5) a face-to-face meeting where the researcher makes a brief presentation and the community experts provide project-specific feedback; (6) compensation of community experts; and (7) a post-meeting summary with additional feedback for the researcher. The CE Studio process has previously been described in detail [12].

## Methods

A retrospective, mixed-methods approach (surveys and interviews) was used to identify and describe the best practices for clinical and translational researchers to engage patients and other community stakeholders through CE Studios. Survey questions included both forced choice and open-ended formats. Interview questions were open-ended to allow for candid responses that would capture the CE Studio experiences and their impact on the researchers, including changes in perception and practices.

*Data Sources* included evaluation surveys completed by both researchers and community experts; a follow-up survey completed by researchers and face-to-face interviews with researchers. The surveys were judged to be exempt by the Vanderbilt IRB. The follow-up survey and interviews were reviewed and approved by the Vanderbilt University IRB.

### Evaluation Survey

In an ongoing effort to improve the quality of our work, every researcher and community expert who participates in a CE Studio completes a brief evaluation survey. The survey includes 16 questions: 11 from the Vanderbilt Clinical and Translational Research Studios evaluation [17] and 4 additional questions specific to the CE Studio. Using a Likert scale, multiple-choice and open-ended questions, the survey assesses the CE Studio process and outcomes and solicits suggestions to improve the process. Respondents rate their satisfaction with the process, indicate their interest in participating in future CE Studios and comment on the community experts’ contribution to the research project. The community experts are asked if the researcher’s presentation gave them enough information to provide feedback and if they felt their input would improve the research. The researchers are asked if their perception about the role of community experts in research had changed and, if so, how it had changed and what they changed in their research project as a result of community input. The intent of this survey is to evaluate the CE Studio program, not to measure specific constructs; therefore, no psychometric testing was conducted.

### Researcher Survey

Researchers were invited to complete a follow-up survey to assess the impact of the CE Studio. The survey consisted of 13 items focusing on what changes they made to their research proposal, research project, or community engagement practices as a result of the input they received from the community experts. The researchers completed the surveys at least 3 months and up to 3 years after their CE Studio. The survey questions were a combination of forced or multiple-choice questions (quantitative) and open-ended questions (qualitative). The surveys were distributed online through REDCap, a secure online web application for surveys and databases, and completed anonymously. As with the evaluation survey, this survey did not measure specific constructs; therefore, no psychometric testing was conducted.

### Researcher Interviews

A total of 10 researchers, selected randomly from follow-up survey respondents, were interviewed about their CE Studio experience. These interviews were conducted 1 month after the researcher surveys were completed. REDCap has a feature that shows who completed the survey without tying their name to their responses. The interviews consisted of 5 open-ended questions about the impact of the community expert input on their attitudes and practices. A graduate student interning with CERC conducted the face-to-face interviews, which took an average of 35 minutes to complete.

### Analysis

Data from the evaluations were analyzed using paired *t*-tests to determine whether researchers and experts assessed the CE Studio experience differently. Data were aggregated by CE Studio using mean values. If no CE Studio was specified, data were aggregated across participants. Independent *t*-tests were used to assess whether experts and researchers differed in the areas they felt experts’ input had contributed to the research project. Data were analyzed using SPSS v24.

Thematic analysis of qualitative survey and interview responses involved an inductive, qualitative content analysis approach to identify emerging themes [18]. Two co-authors performed a line-by-line coding analysis to establish a coding consensus. Key ideas were assigned codes by each co-author and then compared across transcripts for consistency. If any discrepancies arose, they discussed the codes until a consensus was reached. To identify emerging themes while comparing codes, a constant comparison method was used. These findings were confirmed by all the co-authors.

## Results

### Evaluation Survey

Data were analyzed for 75 CE Studios that took place between February 2009 and October 2017. In total, 65 researchers and 591 community experts completed surveys. The average number of community experts that participated in each CE Studio was 8, with a minimum of 3 and a maximum of 12. The researchers that completed the surveys represented a broad range of academic ranks and disciplines. The community experts were diverse in terms of race and ethnicity [48% African-American (n=284), 40% White (n=236), 7% Latino (n=41), and 5% other (n=30), including Asian/Pacific Islander, and Native American] and age [7% 24 or younger (n=41), 36% 25–44 (n=213), 33% 45–64 (n=195), and 24% 65 and older (n=142)]. There were more women (66%, n=390) than men (33%, n=195) and 1% identified their gender as “other” (n=6).

Table 1 shows the average scores of how both community experts and researchers rated their experience with the CE Studio. Thirty of those CE Studios had information (study title) that allowed pairing between the assessments provided by both researchers and experts. As a first step to compare how experts and researchers assessed their experience, we aggregated the data by study title. This resulted in 75 aggregated expert’s observations and 31 researcher’s observations (30 observations where a researcher assessed a CE Studio and 1 aggregated observation across researchers’ surveys where the study title assessed in a CE Studio was not provided). In order to compare how experts and raters assessed their experiences, we performed a paired *t*-test analysis. Results from the paired *t*-tests comparing responses from researchers and community experts to the statement “I was satisfied with the Community Engagement Studio” [scored from “strongly disagree” (1) to “strongly agree” (4)] researchers (mean=3.93, SD=0.26) expressed a higher degree of satisfaction (*t*=3.05, *p*<0.01) than experts (mean=3.73, SD=0.20). To the statement: “The Community Engagement Studio was worth my time” [scored from “too much time” (1) to “not enough time” (3)] researchers (mean=4, SD=0) also expressed a higher appreciation (*t*=8.07, *p*<0.001) for the time spent on the CE Studio than community experts (mean=3.79, SD=0.14).

**Table 1:** Average scores for both researchers and experts on their ratings of the Community Engagement Studio

	Researchers (n=65) [mean (SD)]	Experts (n=591) [mean (SD)]
I was satisfied with the Community Engagement Studio*	3.92 (0.27)	3.70 (0.54)
The Community Engagement Studio was worth my time†	3.98 (0.12)	3.75 (0.51)
The expert feedback was conveyed to me in an appropriate way*	3.89 (0.32)	n/a
This Community Engagement Studio improved the quality of my project*	3.88 (0.33)	n/a
To what degree has the community expert input impacted the patient-centered components of your project? Patient-centered components include patient preferences, patient needs, patient wants and patient values‡	4.50 (0.65)	n/a
The researcher’s presentation gave me enough information to provide appropriate feedback*	n/a	3.60 (0.56)
The feedback provided by the community experts will improve the research project*	n/a	3.67 (0.54)

n/a this question was not included in the survey for either researchers or experts

* From “strongly disagree” (1) to “strongly agree” (4).

† From “too much time” (1) to “not enough time” (4).

‡ From “no impact” (1) to “major impact” (5).

**Fig. 1:**
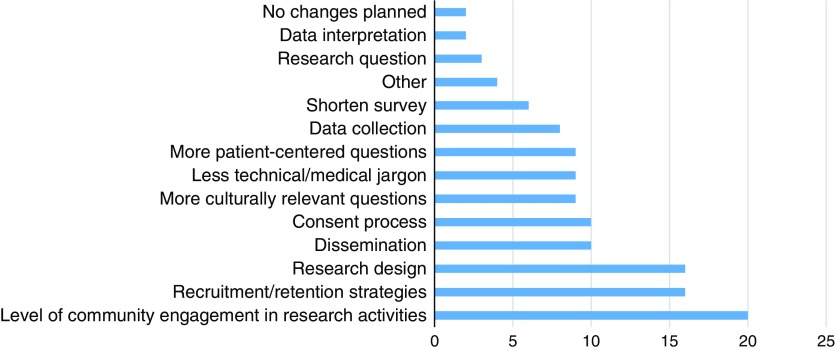
Counts of researchers who checked each possible answer to “What, if anything, do you plan to change as a result of the feedback you received from the Community Engagement Studio?”

We also performed a *t*-test between researchers and experts on how they felt the experts had contributed to the research project in different areas. Table 2 shows the researchers thought that community experts had contributed to increasing their understanding of the community and provided feedback on the appropriateness of the project more than experts thought they had contributed. Fig. 1 shows areas of the research project or process that researchers planned to change after the CE Studio. To the question: “Did your Community Engagement Studio result in any of the following?” results included 20 grant submissions, 1 manuscript submission, 2 delayed grant submissions, and 3 major proposal/manuscript revisions.

**Table 2:** Mean comparison between researcher and expert feelings on the extent of experts’ contribution to the research project

	Researchers’ mean (SD) (n=31)	Experts’ mean (SD) (n=75)	
Increased my/researcher’s understanding of the community	0.86 (0.34)	0.72 (0.19)	2.16*
Increased my/researcher’s sensitivity to the community	0.63 (0.48)	0.58 (0.20)	0.606
Provided feedback on the feasibility of the project	0.70 (0.46)	0.56 (0.19)	1.69
Provided feedback on the appropriateness of the project	0.83 (0.37)	0.52 (0.20)	4.40***
Ideas on recruiting research participants	0.41 (0.49)	0.46 (0.28)	−5.55
Ideas on how to inform the community about the project	0.60 (0.49)	0.53 (0.26)	0.78
Ideas on how to use results of the project to benefit the community	0.57 (0.50)	0.49 (0.20)	0.8
Other	0.13 (0.34)	0.04 (0.08)	1.54

*p<0.05, ***p<0.001 [min-max per item=0 (no)-1 (yes)].

All the researchers (100%, n=65) answered “yes” to the question “Would you request a CE Studio in the future?” and almost all the community experts (99.3%, n=587) answered “yes” to the question “Would you participate in a CE Studio again?” When asked whether they would recommend a CE Studio to a colleague, all the researchers (100%) answered “yes” and 96.7% (n=63) said that they would request input again from the community experts that had participated in that particular CE Studio. To the question on whether the researcher’s perception about the role of patient or community stakeholders in research had changed because of the CE Studio, 63.9% (n=41) researchers said it had not. We did not collect information regarding the nature of their baseline expectations or prior community engagement experience.

The evaluation included 1 open-ended question about ways to improve the CE Studio process. In addition to recommendations about logistics, the themes from the community experts’ responses include adequate preparation to serve in a research advisory role, improving researcher communication, expert diversity, effective facilitation, and follow-up. Similar themes emerged in the researchers’ responses to the same question—expert diversity, effective facilitation, and advanced preparation.

### Researcher Survey

The follow-up survey was sent to the first 39 researchers who participated in a CE Studio and 34 (87%) of them completed the survey. 72.7% (n=25) of the researchers considered it extremely likely they would request a CE Studio again, followed by 18.2% (n=6) who would likely request a CE Studio again and 9.1% (n=3) who were neutral about requesting a CE Studio again. None of the researchers indicated that they would not request a CE Studio again. Over half (56%, n=19) of these researchers have requested a second CE Studio and 12% (n=4) of these have requested a third. These results were in concordance with the evaluation survey results. In response to how satisfied they were with the process, 55.9% (n=19) reported to be extremely satisfied, 38.2% (n=13) were very satisfied, and 5.9% (n=2) were moderately satisfied.

When asked how participation in a CE Studio affected the researchers themselves, 61.8% (n=21) indicated the experience had given them a better understanding of how to address the barriers to participating in research and 76.5% (n=26) indicated that it had given them a better appreciation of the value of patient/community input on research. In addition, 82.3% (n=28) indicated they began taking patient/community values, priorities and concerns into consideration when designing a study. Due to the lack of identifying variables between the surveys, we could not compare responses between the evaluations and follow-up surveys.

### Researcher Interviews

Ten of the 34 researchers who completed the survey were interviewed about the impact of the CE Studio experience on their research attitudes and practices. All 10 respondents (100%) indicated that the experience increased the value they placed on feedback from community stakeholders. They listed specific changes they had made to their project as a result of the feedback (e.g., changes to the research question, design, and recruitment strategies) as well as changes in their attitudes and general approach to community engagement (engaging community members earlier in the research process; seeking more opportunities to engage community in their work; and changing their assumptions about the value of community input). The themes that emerged from a question on their general thoughts about the CES Model resonated with our own experiences and the feedback we have received from community experts. These themes include: the importance of engaging community members early in the research process; the need to prepare or coach researchers before interacting with community; recognition that researchers do not always have the answers and can learn a great deal from community stakeholders; the importance of balancing power between the researchers and the community members to facilitate open and honest discussion; and the importance of having a neutral facilitator to enhance bi-directional communication.

These themes, along with the community expert identified theme “adequate preparation to serve in a research advisory role” inform the practices described below that we believe can enhance the quality of engagement of patients and other community stakeholders in clinical and translational research.

### Early Input

Many of the researchers who responded to our surveys and interview questions indicated that in the future they would seek community input earlier. It is generally accepted that community input can enhance the relevance and success of research through prioritization of research questions, identification of outcomes and comparators relevant to patients, and development of improved strategies to enroll and retain research participants. Our data support the idea that early input, that is, at the idea generation or grant writing stage can help researchers address questions around feasibility and relevance. As one researcher stated: “I wish I had it (the CE Studio) four to five months earlier. Encourage people to go early not late.” Stakeholder input during the course of a study can also help alleviate participant recruitment and implementation problems. Another researcher commented: “What we could have done better is create materials that explain the purpose of the study more coherently. A clearer agenda up front would have been helpful.” Input in the later stages of a research project can inform dissemination plans and subsequent research questions.

### Researcher Coaching

Researchers often have difficulty communicating clearly and appropriately with community members. One community expert noted, “Researchers need to be taught how to culturally and sensitively engage, and listen to nonprofessionals.” Many community experts also commented on the difficulty they had understanding the researchers, suggesting the need for a glossary of technical terms and acronyms and more visual tools to convey key concepts. Researchers also recognized the need for guidance when interacting with community experts. For example, “(*the facilitator*) briefed me in advance about listening which I really needed.”

**Fig. 2:**
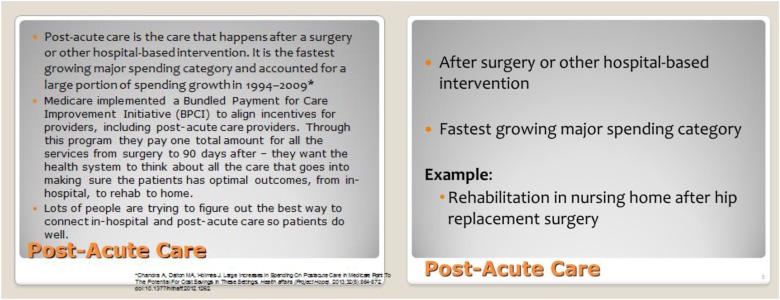
Before and after slide for researcher presentation.

Researchers frequently prepare long presentations that include highly technical language, discipline-specific acronyms, and very complex figures or tables that are text or data dense. To improve communication with community stakeholders, researchers are provided with a template for a brief presentation with instructions to keep text to a minimum and avoid complex tables, formulas, and diagrams. The CE Studio team reviews and may edit the researcher’s slides before their presentation to the community expert panel to make sure the language and images are clear. Fig. 2 compares a researcher’s presentation slide before and after coaching. In this example, much of the text has been moved to the notes section, leaving summary talking points on the “after” slide. This allows the stakeholders to focus on the researcher’s presentation rather than reading a text-dense slide.

Face-to-face coaching is provided during the planning meeting to outline appropriate methods for listening and communicating with a group comprised of community members as opposed to researchers or academicians. This coaching session also walks the researcher through the CE Studio process from beginning to end while sharing tips on verbal communication such as avoiding or clearly explaining acronyms and research jargon. Researchers are also encouraged to limit their comments during the facilitated community expert discussion to clarification questions and simple explanations when called upon by the facilitator. Setting these expectations ensures that the CE Studio remains focused on receiving feedback from the community experts and allowing them to take the lead.

### Researcher Humility

Years of rigorous education and scholarly presentation empowers researchers as experts. To truly benefit from community engagement, researchers must value and respect diverse community knowledge and experiences and be able to listen to, and learn from individuals who are not traditionally considered experts [19]. A theme that emerged in the researcher interviews was that the CE Studio experience led them to realize that they had previously undervalued the input from patients and other community stakeholders. As one researcher stated: “Well, it radically changed, my viewpoint, during the development phase of a research project, the value of input from patients, and … because patient input changed the methodology of my grant.”

In some cases, the community experts felt that the researcher did not value their input, as illustrated by these comments: “I felt the researcher was reluctant to incorporate what we were talking about,” and “I did not feel like the researcher was listening well.” By dropping their preconceived notions about who is and who is not an expert, researchers can better hear and interpret insights from community members. This process allows for a better partnership between the community and researchers.

### Balancing Power

The unequal distribution of power that exists between researchers and stakeholders is a barrier to meaningful communication [11, 19–21]. Researchers’ power comes from their advanced education, academic title, affiliation with an academic institution and professional recognition as a scientist and a substantive authority. Community stakeholders frequently represent groups that have been marginalized in our society and they may not recognize the value of their experiential knowledge. They may also be unfamiliar with institutional norms around meetings such as the right to speak up independently, respectfully disagreeing, speaker participation, and turn-taking style [11, 22–25]. Early CE Studios illustrated that community members may not feel their voice will be heard or that they will not be viewed as experts: “I approached it (*the CE Studio*) with a question about whether we would have power to speak up.” Facilitation techniques to balance power in this setting include inviting and encouraging stakeholders to speak; asking community experts to repeat or explain their comments for clarification and validation; redirecting the conversation when it goes off topic (while respectfully acknowledging the contribution); using body language to show interest in what different experts are saying; and summarizing recurring themes in the discussion. Other tactics to balance the power between researchers and non-researchers include: limiting the number of researchers in the room; dispensing with academic titles and addressing everyone by their first name; having the researcher sit at the table with the community experts rather than stand during the meeting; and limiting the researcher’s comments during the discussion to questions for clarification and responses to direct questions from stakeholders.

### Neutral Facilitator

Utilizing a facilitator who has experience engaging community members and who is not a member of the academic team can help establish that the researcher in the room is a participant and not the person in charge. The most effective facilitators bring experience working with diverse communities that include a broad range of economic class, gender, and ethnicity. They possess a unique skill set that allows them to foster conversation but not dominate it and have a flexible communication style that can adapt to topics ranging from sensitive and traumatic to events that might include routine healthcare visits. The ability to be content neutral is an essential facilitator skill. Establishing rapport and creating a line of open and honest communication is easier when the facilitator is an unbiased individual who does not represent the interests of the researcher or the academic institution. One researcher, when completing the evaluation noted, “Patients feel very safe being honest and open about their thoughts, thanks to the independent, neutral moderator.” Another stated “… the facilitation reduced bias and enhanced objectivity. I imagine this would also elicit more honest responses from the group.” Community experts also noted the importance of the facilitator to keep the conversation on track and to make sure that everyone participated in the conversation.

### Adequate Preparation of Community Stakeholders

Community stakeholders who lack research experience can give meaningful input on the development and implementation of research, but adequate preparation can help connect their experiences to research more effectively. A common theme from the evaluations completed by community experts is the desire to receive information in advance of the CE Studio. “The information ahead of time allowed us to be better prepared. The second time—without preparation, we were struggling to see what we were supposed to respond to.” To prepare stakeholders to be fully engaged in the CE Studio, we provide one-on-one or group orientation and an accompanying guide which outlines the purpose of the CE Studio, the community expert’s role, the CE Studio process and basic information on research (i.e., idea development, design, grant preparation, recruitment, etc.). Experts may also receive an overview of the topic that will be discussed in the meeting or documents to review ahead of time.

## Discussion

Community stakeholders have tremendous potential for informing research by sharing their lived experiences, but this is an area of expertise that is often untapped by researchers. Community stakeholders’ experiential knowledge can improve the quality and relevance of research and enhance research design, implementation, interpretation, and dissemination through the lens of an individual that represents the researcher’s population of interest. The traditional barriers to successful community engagement can be minimized by strategically applying the feedback of community stakeholders and changing researchers’ view of them from passive to active, as engaged users of the research and healthcare enterprises.

Using the structured CE Studio model, we assessed the experiences of 65 researchers and 591 community stakeholders.

A notable finding is that the CE Studios were perceived to be of greater benefit by the researchers as compared to community experts’ perceptions. We believe this points to the need to strengthen our partnership with community experts in the form of bi-directional communication and better follow-up. The CE Studio allows researchers to hear directly from representatives of their population of interest about their lived experience, perceptions and feelings. The community experts do not have a follow-up session to hear how their input directly changed the researcher’s approach. Additionally, a majority (63.9%, n=41) of the researchers completing the evaluation survey reported no change in their perception of the role of community stakeholders in research. The survey is administered immediately after the CE Studio so perceptions may not have changed in this short period. It is also possible that the dose of engagement in the CE Studio is not substantial enough to result in a change in perception.

Some of the themes that emerged from this study reinforce well-established principles of community engagement, including respect, co-learning, and bi-directional communication [13, 26]. We also identified other themes or practices that have not been as well described. These practices are (1) *early input*, that is, seek community input at the idea generation or grant writing phase, (2) *researcher coaching*, to improve their ability to communicate with non-researchers, (3) foster *researcher humility* when engaging the community, (4) *balance power* differences between researchers and stakeholders, (5) utilize a neutral facilitator, and (6) *adequate preparation of community stakeholders* to help them connect their experiences to research. There are many methods for engaging community stakeholders in research and many barriers to doing this effectively. Our findings suggest that utilizing these practices enhances the quality of researcher-stakeholder communication when using the CE Studio model and may be effective with other methods of community engagement.

### Limitations

We did not conduct follow-up surveys and interviews with community experts, so the opinions reflected in the data skews towards the researcher perspective. More investigation is needed on the impact of the CE Studio on the community experts. Additionally, we did not ask the researchers about their experience with community engagement before their CE Studio so we do not know if researchers with community engagement experience would rate their CE Studio experience differently than those who lack community engagement experience. This may have weakened the reported effect of the CE Studio on the researchers’ perceived benefit of the role of the community among those who were not community researchers.

We report data from 65 researchers and 591 experts and we know that some of these researchers and experts participated in more than one studio. However, our survey did not allow the identification of participants so it is possible that we are increasing our risk of type 1 error by treating our sample as independent.

Academic institutions that do not have the infrastructure, personnel, and funds to adequately support community engagement may be limited in their ability to implement the CE Studio and other models of engagement. However, this approach optimizes the use of existing organizational resources so that individual studies do not have to create their own infrastructure. For example, staff that serve in a boundary spanner role (that is, with the ability to communicate with both the researcher and the community) and funds to adequately compensate community experts are essential. This type of established infrastructure ensures that the CE Studio model is implemented effectively and supports the researchers through the process while also identifying and preparing the right stakeholders to participate in the CE Studio.

The CE Studio is not intended for stakeholder engagement that requires a large number of participants or for community-based participatory research. In general, this approach is not recommended if researchers require ongoing stakeholder input. Better approaches for ongoing stakeholder engagement include the creation of a community advisory council or employing stakeholders as consultants or as part of the research team. The CE Studio can help a researcher identify stakeholders or groups that may be interested in forming collaborations for more engaged research. This has been both an intended and unintended outcome of several of our CE Studios as some natural partnerships have formed as a result of the positive interactions between stakeholders and researchers.

## Conclusion

This paper describes our experience with a structured model that can help others develop more effective community engagement programs and more reliably use the CE studio concept. The paper also highlights important practices that should be considered by clinical and translational researchers. We believe these practices—early stakeholder input, researcher coaching, researcher humility, adequate preparation of stakeholders, balancing power and neutral facilitation—can enhance the interaction of researchers and their community partners in a variety of engagement activities. Although there is a large body of literature that establishes best practices for engaging community and patients in research [27–30], many clinical and translational researchers have limited proficiency in this area and many academic institutions do not provide adequate support for mentoring and training in these practices. Future research is needed to test these practices in other approaches to community engagement as well as the best ways to train researchers to incorporate patients and other community stakeholders in their work.
